# Women’s empowerment is associated with maternal nutrition and low birth weight: evidence from Bangladesh Demographic Health Survey

**DOI:** 10.1186/s12905-020-00952-4

**Published:** 2020-05-05

**Authors:** Alamgir Kabir, Md Mahbubur Rashid, Kamal Hossain, Arifuzzaman Khan, Shegufta Shefa Sikder, Heather F. Gidding

**Affiliations:** 1 Centre for Primary Health Care and Equity, Faculty of Medicine, University of New South Wales, Level 3, AGSM Building, Sydney, NSW 2052 Australia; 2 School of Public Health and Community Medicine, Faculty of Medicine, University of New South Wales, Sydney, NSW Australia; 3 Department of Statistics, University of Rajshahi, Rajshahi, Bangladesh; 4 Asian Institute of Disability and Development, Dhaka, Bangladesh; 5 Kala-azar Elimination Program, Dhaka, Bangladesh; 6 School of Public Health, The University of Queensland, Brisbane, Australia; 7 Sexual Reproductive Health and Rights Team, CARE USA, Patna, India; 8 Women and Babies Research, Kolling Institute, Northern Sydney Local Health District, St Leonards, NSW 2065 Australia; 9 The University of Sydney Northern Clinical School, Sydney, NSW Australia; 10 National Centre for Immunisation Research and Surveillance, Westmead, NSW Australia

**Keywords:** Women’s empowerment, Maternal nutrition, Low birth weight, Principal component analysis, Bangladesh, Demographic health survey

## Abstract

**Background:**

The burden of maternal undernutrition and low birth weight (LBW) incurs enormous economic costs due to their adverse consequences. Women’s empowerment is believed to be one of the key factors for attaining maternal and child health and nutritional goals. Our objective was to investigate the association of women’s empowerment with maternal undernutrition and LBW.

**Methods:**

We used nationally representative data from the Bangladesh Demographic Health Survey for 2011 and 2014. We analysed 27357 women and 9234 mother-child pairs. A women’s empowerment index (WEI) was constructed using principal component analysis with five groups of indicators: a) education, b) access to socio-familial decision making, c) economic contribution and access to economic decision making, d) attitudes towards domestic violence and e) mobility. We estimated odds ratios as the measure of association between the WEI and the outcome measures using generalized estimating equations to account for the cluster level correlation.

**Results:**

The overall prevalence of maternal undernutrition was 20% and LBW was 18%. The WEI was significantly associated with both maternal undernutrition and LBW with a dose-response relationship. The adjusted odds of having a LBW baby was 32% [AOR (95% CI): 0.68 (0.57, 0.82)] lower in the highest quartile of the WEI relative to the lowest quartile. Household wealth significantly modified the effect of the WEI on maternal nutrition; in the highest wealth quintile, the odds of maternal undernutrition was 54% [AOR (95% CI): 0.46 (0.33, 0.64)] lower while in the lowest wealth quintile the odds of undernutrition was only 18% [AOR (95% CI): 0.82 (0.67, 1.00)] lower comparing the highest WEI quartile with the lowest WEI quartile. However, the absolute differences in prevalence of undernutrition between the highest and lowest WEI quartiles were similar across wealth quintiles (6–8%).

**Conclusions:**

This study used a comprehensive measure of women’s empowerment and provides strong evidence that low levels of women’s empowerment are associated with maternal undernutrition as well as with delivering LBW babies in Bangladesh. Therefore, policies to increase empowerment of women would contribute to improved public health.

## Background

About half of the world’s population is affected by maternal and child under-nutrition [1, 2]. Undernourishment of women in reproductive age is more common in South Asia than any other region [3]. In the South Asian region, prevalence of maternal undernutrition varies between 10 and 40% [1]. Particularly in Bangladesh, the prevalence of undernutrition among females is much higher than any other developing country, [3] with more than 30% women of reproductive age reported to be malnourished [4]. Maternal under-nutrition has persistently been reported to be a major contributor to morbidity, mortality and poor birth outcomes including low birth weight (LBW), neonatal mortality, and subsequent childhood undernutrition [1]. Maternal undernutrition alone accounts for about 25–50% of intrauterine growth restriction [5]. In such a way, undernutrition can transfer from one generation to other.

Globally, about 20.6 million children are born with a low birth weight (LBW) each year. Among them, 96.5% are from low and middle income countries (LMICs) while the global estimate of LBW prevalence is 15.5% [6]. The prevalence of LBW significantly varies across the United Nations regions, such as South-central Asia has the highest incidence of LBW (27%) and the lowest in Europe (6.4%) [6]. In rural Bangladesh around 55% babies are born with LBW [7]. However, the national survey of Bangladesh reported the prevalence of LBW as 36% [8]. The consequences of LBW are universally recognized. For example, it reportedly contributes to child mortality, [9] undernutrition, [10] long term disability and impaired development, [11] shorter adult height, [10] delayed motor and social development, [12] having a lower IQ [10]. Consequently, LBW incurs enormous economic costs, higher medical expenditures, special education and social service expenses and decreased productivity in adulthood.

Maternal undernutrition is caused by multiple factors in developing countries. Women from the developing countries lag behind men in having access to food, health care and education [13]. A study from Bangladesh reported that women’s education, exposure to media, and domestic decision-making status significantly influenced the nutritional status of women [14]. Another study reported similar results: female literacy, poverty and lack of empowerment were the major barriers to improving maternal nutrition in South Asia [5]. Other variables that also increase the likelihood of maternal undernutrition, include various biologic and social stresses, such as food insecurity and inadequate diet, recurrent infections, poor health care, heavy work burdens, and gender inequities [14, 15].

**Fig. 1:**
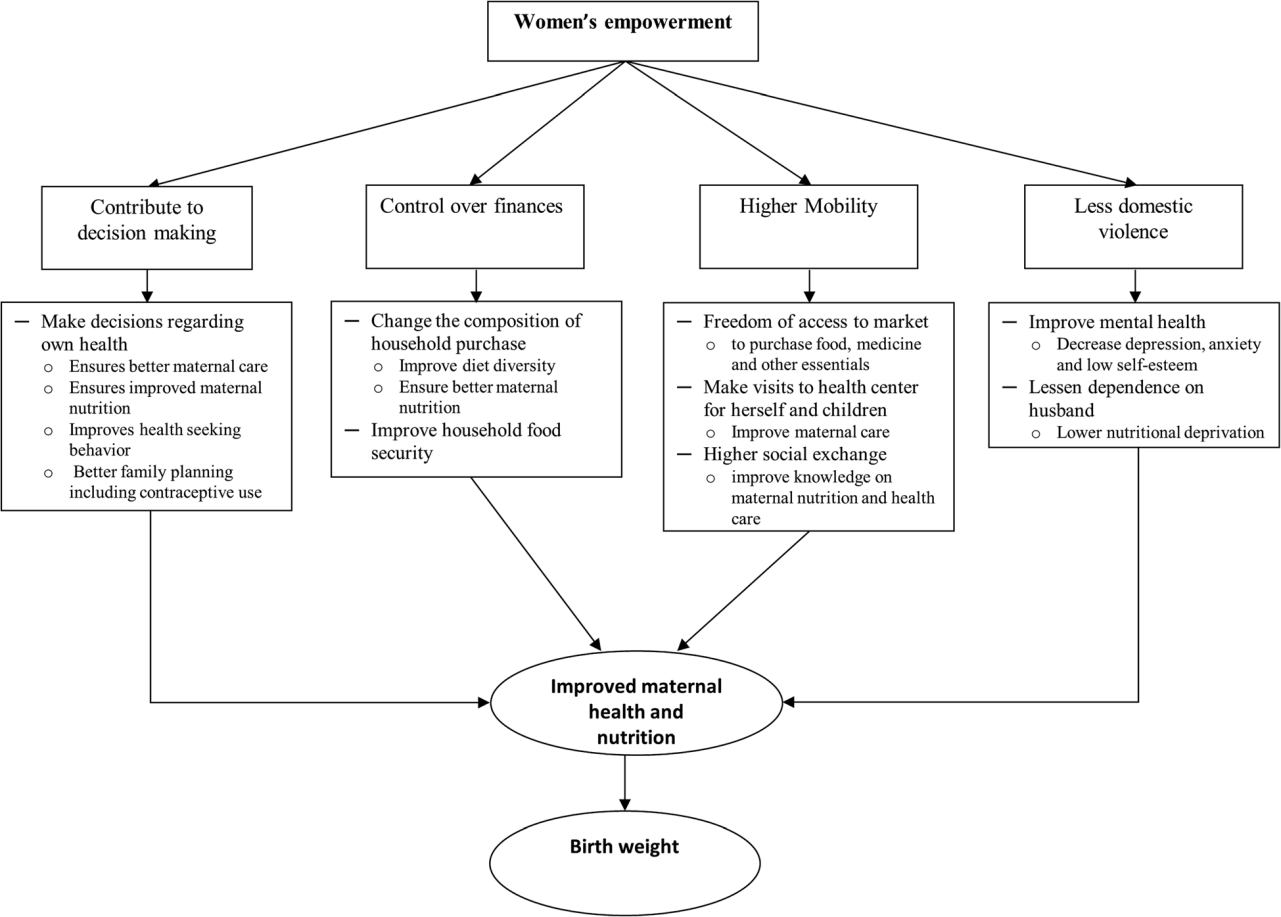
Conceptual framework

Women’s empowerment, which is believed to be one of the key factors for attaining maternal and child health and nutritional goals [16], can influence all the factors associated with maternal nutritional status to some extent. The pathway of how the empowerment of women affects maternal nutritional status and birth weight is described in Fig. 1. Empowered women have the ability to control decision-making in different aspects of life which include socio-cultural, familial and interpersonal and legal dimensions [17, 18]. They can independently make decisions about their own health as well as their children’s health. As a result, women’s empowerment can ensure better maternal care, improved maternal nutrition, and provide freedom in choosing healthy family planning methods. Empowered women have control over finances. Thus, they can change the composition of household purchases, which improves household food security as well as the diet diversity and nutritional status of both themselves and their children [19–22]. They can also allocate more money for the education and health of their family [23]. Empowered women have higher mobility, which increases their freedom to visit food markets and attend health center appointments for both herself and for her children and visit friends or relatives. As a result, they acquire resources such as information and support [24] which help to improve maternal and child health care. Finally, empowerment of women has been reported to lessen the risk of domestic violence [25] which contributes to improving maternal mental health [26] and lowering maternal nutritional deprivation [3]. Studies from LMICs reported that women’s empowerment has a significant influence on child nutrition, [27–29] infant and young child feeding, [24, 28] reproductive health, [17, 30] health seeking behavior [23] and maternal health service utilization [31]. Therefore, the impact of maternal undernutrition on the health of children throughout their life is considered irreversible [32, 33].

While many studies have been conducted in LMICs to investigate the association between women’s empowerment and various health outcomes, the indicators used to define empowerment remain elusive. There are many different indicators, used to define women’s empowerment, available in the literature [18, 19, 24, 34, 35] which entail that empowerment is a dynamic process of change by which “those who have been denied the ability to make choices acquire such an ability” [34]. However, a comprehensive measure of women’s empowerment is lacking. Due to its latent phenomena, different studies used different indicators to measure women’s empowerment [36]. A recent study suggested some indicators to construct a survey-based women’s empowerment index (SWPER) in Africa [37] to measure progress towards the Sustainable Development Goal 5: achieving gender equality and empower all women and girls [38]. However, there is no scientific consensus on which indicators should be used or how to weigh them to construct a women’s empowerment index. Studies conducted to date using Demographic Health Surveys (DHS) to measure women’s empowerment have generally used two types of indicators: household decision-making and attitudes to wife beating [24, 39]. However, there are other potentially important indicators in the DHS data set that could be used, as proposed in other studies [36] such as participation in a microcredit programme (membership of Non-Government Organization, NGO) and education. To our knowledge, a very few studies investigating women’s empowerment have taken into account the covariation among the indicator variables when constructing a women’s empowerment index [23, 24, 31, 36, 39]. Furthermore, the few studies examining the association between women’s empowerment and maternal and child undernutrition are not consistent [27]. For example, a study from Benin [40] and other one from Nepal [41] suggested that women’s empowerment is significantly associated with maternal nutritional status, however, another study from Ghana [42] found no association. Similarly, Begum and Sen (2009) [43] found no association between women’s empowerment and child’s nutrition in Bangladesh, but another study from India [44] reported a significant association. Another study reported that there is a direct link between women’s empowerment and premature delivery, [45] which is one of the key factors affecting birth weight. However, there is an inadequate number of studies to investigate the association between women’s empowerment and birth weight. Therefore, we aimed to develop a comprehensive indicator for empowerment of women using principal component analysis (PCA) methods to account for the covariation among the indicator variables and assess the association of the index with maternal undernutrition and LBW using Bangladesh Demographic Health Survey (BDHS) data.

## Methods

### Data source

We used nationally representative data from the BDHSs conducted in 2011 and 2014 to maximize the sample size and to be able to construct a women’s empowerment index (WEI) across the two time points. Both surveys were nationally representative cross-sectional surveys based on a two-stage stratified sample of households. The details of the survey design are described in detail elsewhere [4, 46]. In brief, the first stage sample is of 600 enumeration areas (EAs), 207 from urban and 393 from rural areas, selected with a probability proportion to size from a list of EAs across Bangladesh (generated by the Bangladesh Bureau of Statistics during the Population and Household Census in 2011). On average, each EA consists of about 120 households in both surveys which served as a sampling frame for the second stage sampling. In the second stage sampling, on average about 30 households were selected systematically with equal probability of selection from each selected EA. In order to prevent bias, no replacement and or changes to the pre-selected households were allowed. Data collection for the 2011 survey was conducted in five phases between July and December and for the 2014 survey four phases were conducted between June and November. The inclusion criteria for our study were women who were (i) currently married, (ii) currently living with their husband and (iii) currently sexually active (in the 4 weeks preceding the survey, they either had sex at least once with their partner or did not have sex due to postpartum abstinence). We set these inclusion criteria as we presumed that the responses on the women’s empowerment indicators, described in the following section, would have been different between women who hold and who did not hold these criteria. Therefore, with 18000 households selected in each survey there were an expected 18000 ever-married women available to include in our study.

### Indicators used for women’s empowerment index construction

The survey data were collected using structured questionnaires. Data collected included household characteristics, demographic characteristics of the household members, anthropometry of both the women and their children under 5 years of age, social characteristics and reproductive history of the women, treatment seeking behavior, husband’s socio-demographic characteristics, woman’s contribution to running the household and attitudes to violence, child’s immunization status, and HIV/AIDS diagnoses. To construct the WEI we used most of the indicators proposed by Ewerling et al. (2017) [37] and additional indicators used in other studies [23, 24, 27, 43]. We constructed the WEI as a composite of five groups of indicators: a) education, [27, 37] b) access to socio-familial decision making (contraception use, woman’s health care, children’s health care, and relative’s home visit), [23, 24, 37, 43] c) economic contribution and access to economic decision making (spending of their own earnings, ability to purchase large house items, and NGO membership), [23, 24, 37, 43] d) attitudes towards domestic violence (physical violence justified in the following situations: if the women goes outside without informing her husband, neglects her children, argues with husband, and refuses to have sex), [24, 37] and e) mobility (visits health center alone) [23, 24, 43]. All the indicator variables were categorized into ordinal variables. Education was classified into four-ordered categories as no education (0), primary (1), secondary (2) and higher secondary or more (3). All of the indicator variables for decision making were categorized into three or four ordered categories (0 = not eligible for making any decision, e.g. women who never used contraception were not asked about who made decisions about choosing contraception or women who were unemployed were not asked about who made decisions on spending their earnings; 1 = husband or other, 2 = jointly with husband and 3 = women herself) and the variable for mobility (visit health center alone) was categorized into three ordered categories (0 = never visited health center, 1 = along with other and 2 = alone).

### Outcome variables

In this study, there were two outcome variables. The first was maternal undernutrition which was defined as body mass index (BMI) < 18.5 [1]. BMI was calculated as weight, in kg, divided by squared height in meters. Weight of the women was measured in kilograms using Seca digital scale and height was measured in centimeters using a Shorr height board by the trained anthropometrist [47]. The other outcome variable was low birth weight (LBW) which was defined based on the mother’s perception of the size of their last-born baby within the last 3 years of interview as the actual birth weight is not available in the demographic health survey. Many studies have already established that mother’s perception of birth size is a good proxy for birth weight in large nationally representative surveys [48, 49]. Women’s perception was categorized into five groups: very large, larger than average, average, smaller than average and very small. For the purposes of the analysis, we defined LBW as a binomial variable – LBW = 1 if birth size was smaller than average or very small and LBW = 0 otherwise.

### Potential confounders

Women and their husband’s educational qualifications were categorized as described above. Women’s employment status was categorized as currently working at the time of interview and not working. The wealth index was provided as part of the demographic and health survey dataset, and was constructed using PCA as described elsewhere [50]. The wealth index was classified into quintiles. Presence of a sanitary toilet was defined as a household having a latrine with any type of flush or pit toilet latrine or ventilated improved pit latrine or pit latrine with slab.

### Statistical analysis

For WEI construction, we applied PCA, which is a validated and widely accepted method for constructing indices [51–53]. PCA is a multivariate statistical method that transforms a number of (correlated) variables into a smaller number of uncorrelated variables called principal components. The first principal component explains as much of the variability in the data as possible, and each successive component explains as much of the remaining variability as possible. Before performing PCA, all the indicator variables were centered at zero and scaled to unit variance. With all the indicator variables in the model, the first principal component was regarded as the WEI. For validation, we used boxplots to compare the distribution of the WEI for each category of the variables used in the WEI construction. The WEI was further categorized into 4 quartiles to assess the dose-response relationship with maternal undernutrition and birth weight of their last-born baby. To compare the characteristics of women, their household and their children by maternal nutritional status (under-nourished vs well-nourished) and between low and normal birth weight babies, we used chi-squared test for categorical variables, t-test for normally distributed continuous variables and the Mann-Whitney U test for non-normal continuous variables. We estimated odds ratios (OR) as the measure of association between the WEI and the two outcome measures using generalized estimating equations (GEE) with a logit link and exchangeable correlation structure to account for the cluster (enumeration area) level correlation. We obtained 95% confidence intervals and *p*-values from the GEE model. Potential confounders which were associated with the outcome variables at *p* < 0.20 in the univariate analysis were adjusted for by including them in a multivariable model. We set *p* < 0.05 for statistical significance. We also examined the interaction of WEI with wealth quintile on maternal undernutrition and birth weight to see whether the impact of WEI on maternal nutrition and birth weight varied by wealth quintile. Data management and analyses were conducted with statistical software, R version 3.3.3.

## Results

**Fig. 2:**
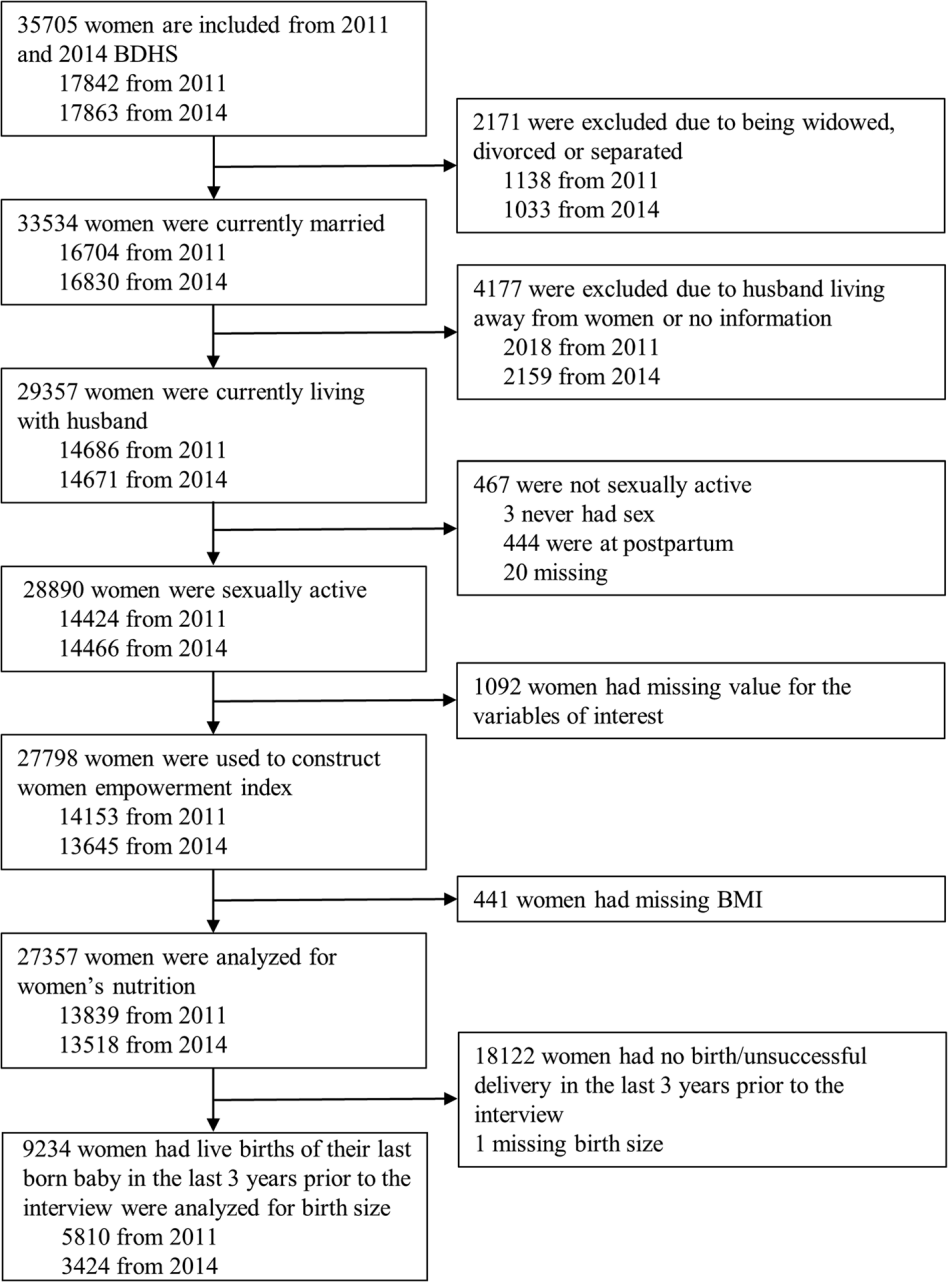
Assembling the study population from Bangladesh demographic health survey (BDHS) in 2011 and 2014

**Fig. 3:**
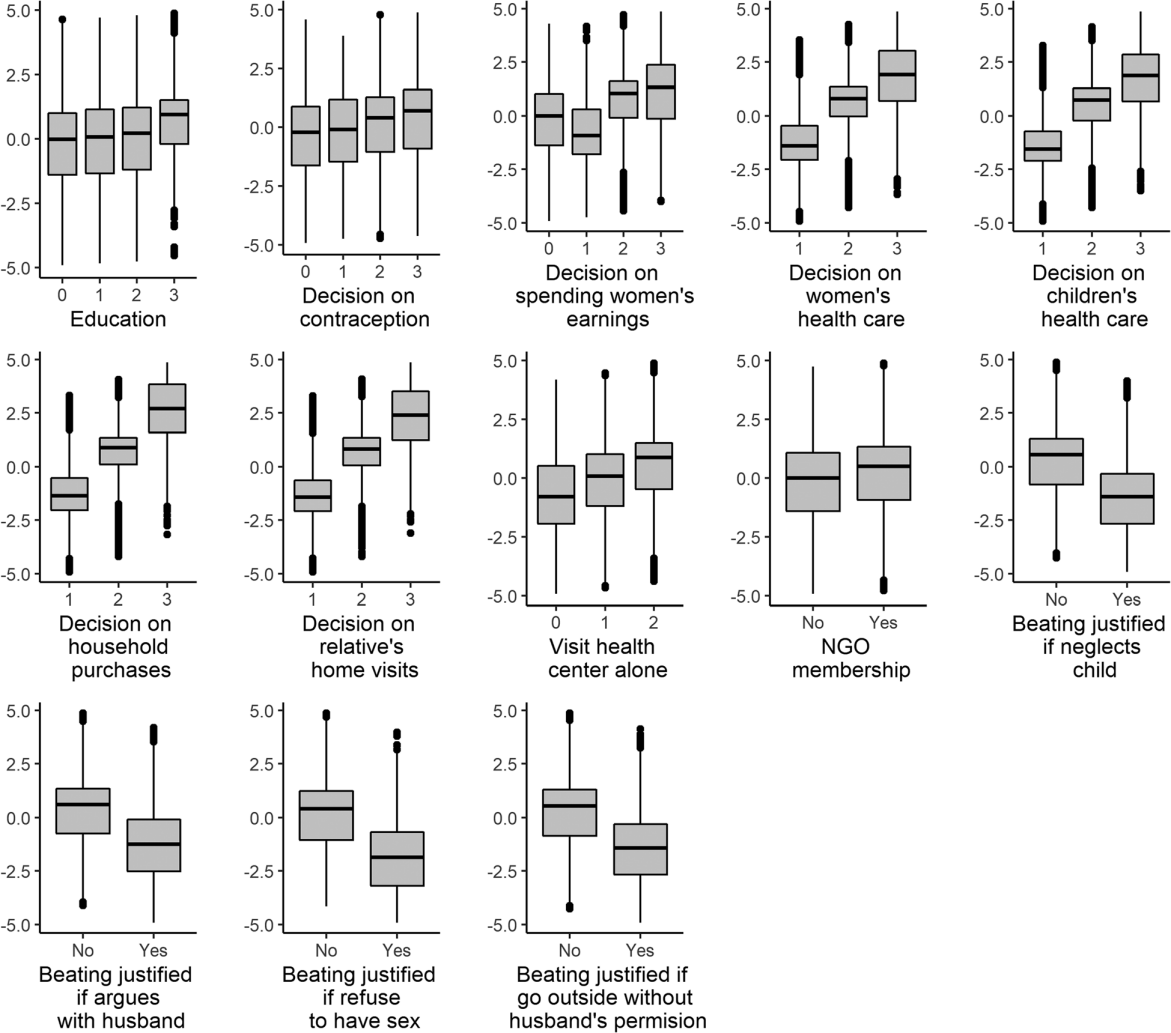
Validation of women’s empowerment index (WEI) construction: distribution of WEI at each point of the variables used to construct WEI

Of the 35705 married women of reproductive age interviewed, 27798 (78%) women met the inclusion criteria for WEI construction (Fig. 2). We analyzed 27357 women for the association between WEI and maternal undernutrition and 9234 women-child pairs to assess the association between WEI and LBW. The age range of the women was 13–49 years and 10.6% were adolescent, i.e. ≤19 years of age (data not shown). The first principal component of the WEI explained 21% of the total variation of all the indicators used to construct the index (data not shown). The box plots (Fig. 3) display the distribution of the WEI for each category of each variable used to construct the WEI. All of the box plots show that the WEI constructed using PCA maintained the order of the variable’s categories; that is the higher the category the higher WEI.

Characteristics were compared between well-nourished and malnourished women and between the LBW and normal birth weight (NBW) babies (Table 1). The overall prevalence of maternal undernutrition was 20% (5483/27357). All characteristics were statistically significantly (*p* < 0.001) associated with maternal undernutrition status. Women with undernutrition and their husbands were more likely to be less educated than their counterparts. Malnourished women were more likely to come from the lower wealth quintiles. Rural residency was higher among malnourished women compared to well-nourished women. Households of malnourished women were less likely to have sanitary toilets than that of the well-nourished women. The prevalence of LBW was 18% (1679/9234). Maternal age, working status, parity, rural residency and the year of interview were comparable between LBW and NBW babies. Mothers of LBW babies were more likely to be malnourished than mothers of NBW babies (*p* < 0.001). Parents of LBW babies had less education compared with the parents of NBW infant (*p* < 0.001). Low birth weight was more prevalent among female babies (*p* < 0.001). The presence of sanitary toilets was less common among the households of the LBW babies (*p* < 0.001).

**Table 1:** Participants characteristics by maternal nutritional and low birth weight status

Characteristics	Maternal nutritional status			Birth Weight		
	Under-nourished (BMI < 18.5)	Well-nourished (BMI ≥ 18.5)	*p*-value	Low birth weight (LBW)	Normal birth weight (NBW)	*p*-value
	*n* = 5483	*n* = 21874		*N* = 1679	*N* = 7555	
**Women’s age, Mean (SD) yrs**	29.5 (9.6)	31.4 (9.0)	< 0.001	25.3 (6.3)	25.4 (6.0)	0.694
**Maternal Undernutrition n (%)**	–	–		528 (31.4)	1898 (25.1)	< 0.001
**Women’s education, n (%)**						
No education	1754 (32.0)	5070 (23.2)		358 (21.3)	1269 (16.8)	
Primary	1862 (34.0)	6498 (29.7)		547 (32.6)	2234 (29.6)	
Secondary	1699 (31.0)	8176 (37.4)		670 (39.9)	3349 (44.3)	
Higher	168 (3.1)	2130 (9.7)	< 0.001	104 (6.2)	703 (9.3)	< 0.001
**Currently working women, n (%)**	1002 (18.3)	4621 (21.1)	< 0.001	233 (13. 9)	1083 (14.3)	0.662
**Husband’s education, n (%)**						
No education	2174 (39.7)	5883 (26.9)		535 (31.9)	1978 (26.2)	
Primary	1762 (32.1)	5947 (27.2)		531 (31.6)	2270 (30.1)	
Secondary	1237 (22.6)	6451 (29.5)		435 (25.9)	2223 (29.4)	
Higher	309 (5.6)	3586 (16.4)	< 0.001	177 (10.5)	1079 (14.3)	< 0.001
**Parity, median (IQR)**	2 (1, 4)	2 (1, 3)	< 0.001	2 (1, 3)	2 (1, 3)	0.939
**No. of antenatal visits, Median (IQR)**	–	–	–	1 (0, 3)	2 (0, 4)	< 0.001
**Undesired pregnancy, n (%)**	–	–	–	525 (31.3)	2155 (28.5)	0.027
**Female infant, n (%)**	–	–	–	908 (54.1)	3565 (47.2)	< 0.001
**Wealth quintiles, n (%)**						
Quintile 1 (Lowest)	1711 (31.2)	3363 (15.4)		441 (26.3)	1633 (21.6)	
Quintile 2	1370 (25.0)	3917 (17.9)		370 (22.0)	1466 (19.4)	
Quintile 3	1101 (20.1)	4262 (19.5)		298 (17.7)	1423 (18.8)	
Quintile 4	860 (15.7)	4714 (21.5)		297 (17.7)	1500 (19.8)	
Quintile 5 (Highest)	441 (8.0)	5618 (25.7)	< 0.001	273 (16.3)	1533 (20.3)	< 0.001
**Rural residency, n (%)**	4177 (76.2)	13459 (61.5)	< 0.001	1153 (68.7)	5023 (66.5)	0.090
**Year of interview, n (%)**						
2011	3136 (57.2)	10703 (48.9)		1026 (61.1)	4783 (63.3)	
2014	2347 (42.8)	11171 (51.1)	< 0.001	653 (38.9)	2772 (36.7)	0.097
**Household had sanitary toilet, n (%)**	2674 (52.2)	14418 (69.9)	< 0.001	873 (56.3)	4294 (62.0)	< 0.001
**Toilet shared with other household, n (%)**	1897 (39.4)	6534 (32.6)	< 0.001	624 (42.2)	2577 (38.6)	0.011

Missing value: currently working women (*n* = 1 for maternal nutritional status & *n* = 1 for birth weight), Husband’s education (*n* = 8 for maternal nutritional status & *n* = 6 for birth weight), No. of antenatal visits (*n* = 10 for birth weight), undesired pregnancy (*n* = 1 for birth weight), household had sanitary toilet (*n* = 1612 for maternal nutritional status & *n* = 763 for birth weight) and toilet shared with other household (*n* = 2490 for maternal nutritional status & *n* = 1076 for birth weight)

There was a significant interaction (*p* < 0.05) between household wealth quintile and WEI when examining the outcome of maternal undernutrition. Therefore, we presented a stratified analysis for maternal undernutrition by wealth quintiles (Table 2). The stratified analysis by household wealth quintiles suggested that the association between increasing WEI and decreasing undernutrition was strongest in the highest quintile (Quintile 5) of wealth. In the highest wealth quintile, the odds of undernutrition was 54% [AOR (95% CI): 0.44 (0.33, 0.64)] lower in the highest (fourth) quartile of WEI compared with the lowest (first) quintile. In the lowest wealth quintile (Quintile 1), no significant association between women’s empowerment and maternal undernutrition was observed. Even though the relative difference was highest.

**Table 2:** Maternal undernutrition prevalence by quartile of women’s empowerment index (WEI) and relative odds of being undernourished

WEI quartile	N	Undernutrition n (%)	OR (95% CI)	*p*-value	AOR^a^ (95% CI)	*p*-value
**Wealth quintile 1 (Lowest)**						
Quartile 1 (Lowest)	1534	542 (35.3)	1		1	
Quartile 2	1365	472 (34.6)	0.96 (0.80, 1.13)	0.599	0.97 (0.82, 1.16)	0.773
Quartile 3	1228	415 (33.8)	0.93 (0.79, 1.10)	0.415	0.95 (0.80, 1.13)	0.544
Quartile 4 (Highest)	947	282 (29. 8)	0.79 (0.65, 0.96)	0.020	0.82 (0.67, 1.00)	0.054
p for trend	–	0.090	0.067	–	0.087	–
**Wealth quintile 2**						
Quartile 1 (Lowest)	1496	448 (30.0)	1		1	
Quartile 2	1397	385 (27. 6)	0.87 (0.73, 1.04)	0.134	0.89 (0.75, 1.06)	0.172
Quartile 3	1344	308 (22.9)	0.72 (0.60, 0.86)	< 0.001	0.74 (0.62, 0.89)	0.001
Quartile 4 (Highest)	1050	229 (21.8)	0.65 (0.53, 0.80)	< 0.001	0.68 (0.56, 0.84)	< 0.001
p for trend	–	0.024	0.010	–	0.009	–
**Wealth quintile 3**						
Quartile 1 (Lowest)	1475	356 (24.1)	1		1	
Quartile 2	1361	283 (20.8)	0.82 (0.68, 0.98)	0.033	0.85 (0.70, 1.02)	0.085
Quartile 3	1331	262 (19.7)	0.79 (0.65, 0.96)	0.019	0.86 (0.70, 1.04)	0.123
Quartile 4 (Highest)	1196	200 (16.7)	0.64 (0.52, 0.78)	< 0.001	0.72 (0.58, 0.88)	0.001
p for trend	–	0.016	0.030	–	0.065	–
**Wealth quintile 4**						
Quartile 1 (Lowest)	1329	263 (19.8)	1		1	
Quartile 2	1401	219 (15.6)	0.76 (0.62, 0.94)	0.011	0.82 (0.66, 1.01)	0.067
Quartile 3	1393	191 (13.7)	0.67 (0.54, 0.83)	0.000	0.75 (0.60, 0.93)	0.010
Quartile 4 (Highest)	1451	187 (12. 9)	0.63 (0.51, 0.77)	0.000	0.75 (0.60, 0.93)	0.011
p for trend	–	0.052	0.066	–	0.136	–
**Wealth quintile 5 (Highest)**						
Quartile 1 (Lowest)	1015	119 (11.7)	1		1	
Quartile 2	1313	119 (9.1)	0.70 (0.53, 0.92)	0.010	0.78 (0.58, 1.03)	0.080
Quartile 3	1641	114 (7.0)	0.55 (0.42, 0.73)	0.000	0.72 (0.54, 0.96)	0.026
Quartile 4 (Highest)	2090	89 (4.3)	0.31 (0.23, 0.42)	0.000	0.46 (0.33, 0.64)	< 0.001
p for trend	–	0.001	0.008	–	0.022	–

^a^*AOR* Adjusted odds ratio, adjusted for age, husband’s education, parity, rural residency, year of interview, household sanitary toilet and toilet shared with others in the highest wealth quintile, the absolute differences in prevalence of undernutrition between the highest and lowest WEI quartiles were similar across the wealth quintiles (6–8%).

The prevalence of LBW declined from the lowest to the highest quartile of WEI in a dose response manner (Table 3). While comparing with the first quartile of WEI, the odds of having LBW was 32% [AOR (95% CI): 0.68 (0.57, 0.82)] lower in the 4th quartile, 21% [AOR (95% CI): 0.79 (0.68, 0.93)] lower in the 3rd quartile, and only 9% [AOR (95% CI): 0.91 (0.78, 1.06)] lower in the 2nd quartile. This decreasing trend of relative odds was statistically significant (*p* < 0.001 for linear trend).

**Table 3:** Prevalence of low birth weight (LBW) by the quartiles of women’s empowerment index (WEI) and the relative odds of having LBW

WEI quartile	N	LBW n (%)	OR (95% CI)	*p*-value	AOR^a^ (95% CI)	*p*-value
Quartile 1 (Lowest)	2546	548 (21.5)	1		1	
Quartile 2	2442	459 (18.8)	0.89 (0.77, 1.04)	0.139	0.91 (0.78, 1.05)	0.201
Quartile 3	2348	403 (17.2)	0.77 (0.66, 0.90)	0.001	0.79 (0.68, 0.93)	0.004
Quartile 4 (Highest)	2042	297 (14.5)	0.65 (0.55, 0.77)	< 0.001	0.68 (0.57, 0.82)	< 0.001

^a^*AOR* Adjusted odds ratio, adjusted for maternal undernutrition, paternal education, no of antenatal visit, undesired pregnancy, female infant, wealth quintiles, rural residency, year of interview, household sanitary toilet and toilet shared with others

## Discussion

This study found a significant association between women’s empowerment and both maternal undernutrition and low birth weight using nationally representative data from the BDHS. The likelihood of being malnourished or delivering a LBW baby reduced with increasing WEI. Household wealth significantly modified the association between women’s empowerment and maternal undernutrition; the association was stronger in the highest quintile of the wealth index. On the other hand, increases in WEI led to similar absolute reductions in prevalence of undernutrition regardless of wealth quintile. As the burden of maternal undernutrition and low birth weight are high in lower- and lower-middle income countries, the benefit of improving women’s empowerment at a population level is likely to be considerable.

Our findings are consistent with other studies examining the association between women’s empowerment and undernutrition even though different WEI indicators were used. A recent study investigated the association between agriculture-based women’s empowerment and dietary quality among household members in Rural Bangladesh [54]. The authors found a significant positive association between women’s empowerment and the adult men’s and women’s dietary diversity and nutrient intake [54]. Therefore, it can be said that women’s empowerment in agriculture is associated with increased BMI mediated through diverse food and nutrition intake [55] which supports our study finding that women’s empowerment is associated with a lower odds of maternal undernutrition. Another cross-sectional study from a rural area of Nepal investigated the association between women’s empowerment in agriculture and maternal nutrition and reported a positive association with maternal BMI [41]. Two cross-sectional studies from low- or lower-middle-income countries in Africa also reported a positive association between women’s empowerment and maternal nutrition: one used similar indicators for WEI [40] to ours and the other one used agriculture-based indicators to measure WEI [42]. Although the study from Ghana found no significant association between women’s empowerment and maternal nutrition or child nutrition, [42] the direction of association was similar to ours.

In contrast to previous studies, our study found that household wealth status modified the effect of women’s empowerment on maternal nutrition. Therefore, future studies should consider household wealth status when measuring the association of women’s empowerment and maternal undernutrition. The highest wealth quintile had the highest relative association and this can be explained by the low overall prevalence of undernutrition: 11.7% in the lowest and 4.3% in the highest WEI quartiles. However, the prevalence of undernutrition in the lower wealth quintiles was considerably higher (35.3% in the highest and 29.8% in the lowest WEI quartiles) and if we look into the absolute differences, women’s empowerment reduced maternal undernutrition to the same degree irrespective of wealth quintile. Therefore, although the relative association is not statistically significant in the lower wealth quintiles, the association is clinically meaningful in regard to reducing overall burden of undernutrition at the population level. So, improving women’s empowerment irrespective of the household wealth status would have a considerable impact on reducing undernutrition in women in countries with a high burden such as Bangladesh.

The association between high WEI and LBW has also been reported previously. A study from rural Bangladesh evaluated the effect of women’s decision making autonomy on infant’s birth weight using 6 indicator variables [56]. The authors reported that women with the lowest (1st tertile) autonomy had a 40% higher risk of having a LBW infant compared to women with the highest (3rd tertile) autonomy. Although this study did not represent the whole of Bangladesh and used fewer indicators than ours, it provides support to our study findings in terms of both direction and magnitude. Two studies from India also reported that indicators of women’s autonomy were significantly associated with LBW [57] with one reporting that high women’s autonomy was associated with a 18% lower risk of LBW compared to the low autonomy [58]. An intervention study conducted in Mexico in 1997 provided incentives, training and information to the poor women to make them empowered [59] and found a significant reduction in LBW (44.5%) and improved quality of prenatal care [59]. Although we used survey-based indictors to construct a WEI, our results are consistent with this intervention study.

The main strength of this study is that it used comprehensive population-based measures of women’s empowerment in a South Asian population. We also considered household wealth status when measuring the association of women’s empowerment with maternal undernutrition. Another advantage of this study is that it used PCA methods which assigned weights to each of the variables by taking into account the covariation between the indicator variables [37, 60]. So, we believe this study provides more valid and reliable estimates than previously published studies and thus provides important evidence that women’s empowerment is a key driver of maternal and child nutrition.

Limitations of this study may include potential residual confounding and information bias inherent in conducting a secondary analysis of survey data. About 10% women in our study were adolescent and WHO recommended to use z-score as a measure of nutritional status. As we used BMI as the measure of maternal nutritional status, the malnutrition prevalence could be underestimated. To define LBW we used maternal perception of birth size (by asking question “was the newborn very large, larger than average, average, smaller than average or very small?”) as a proxy for birth weight. We found the prevalence of LBW to be only 18% which is much lower than the 55% reported from rural Bangladesh [61, 62] and 36% nationally [63] suggesting some misclassification. The perception might also have varied between the maternal education and socio-economic status categories, although the participants were unaware of the study outcomes and thus non-differential misclassification bias may have occurred which could have led to an underestimation of the true associations. Due to probability sampling, there is a chance that a woman could be selected in both surveys. However, based on our calculation (1 in 3.9 million women), we believe this is very unlikely.

## Conclusions

Women’s empowerment is considered to be a key driver for attaining maternal and child health and nutritional goals. Our findings provide evidence that empowerment of women has a significant association with maternal undernutrition as well as LBW in Bangladesh. They suggest that policies to increase empowerment of women would contribute to improve public health. However, a standard guideline is needed to measure women’s empowerment for future studies in this context as suggested by Ewerling et al. (2017) for the African population [37].

## Data Availability

The datasets supporting the conclusions of this article are freely available online in https://dhsprogram.com/data/available-datasets.cfm.

## Abbreviations

LBW: Low Birth Weight
NBW: Normal Birth Weight
WEI: Women’s Empowerment Index
OR: Odds Ratios
AOR: Adjusted Odds Ratio
LMIC: Low and Middle Income Country
SWPER: Survey-Based Women’s Empowerment Index
DHS: Demographic Health Surveys
BDHS: Bangladesh Demographic Health Survey
NGO: Non-Government Organization
PCA: Principal Component Analysis
EA: Enumeration Areas
BMI: Body Mass Index
GEE: Generalized Estimating Eqs.
CI: Confidence Interval
